# Valorization of Bayberry (*Morella rubra*) Leaf By-Products: Impact of Growth Stage and Drying Method on Phytochemical Profile and Potential as Functional Food Ingredients

**DOI:** 10.3390/plants15060945

**Published:** 2026-03-19

**Authors:** Yoko Tsurunaga, Tomoyoshi Hara, Yasuo Oowatari, Masatomo Makino, Junko Kasuga, Shingo Matsumoto

**Affiliations:** 1 Faculty of Human Science, Shimane University, Matsue 690-8504, Japan; h231049@matsu.shimane-u.ac.jp; 2 Graduate School of Human and Social Sciences, Shimane University, Matsue 690-8504, Japan; 3 The United Graduate School of Agricultural Sciences, Tottori University, 4-101 Koyama-Minami, Tottori 680-8553, Japan; smatsu@life.shimane-u.ac.jp; 4 Shimane Institute for Industrial Technology, 1 Hokuryo-cho, Matsue 690-0816, Japan; 5 Faculty of Life and Environmental Science, Shimane University, Matsue 690-8504, Japan

**Keywords:** myricitrin, polyphenols, antioxidant activity, freeze-drying, volatile profile, peltate glandular trichomes, GC-MS, LC-MS/MS

## Abstract

Bayberry (*Morella rubra* Lour.; syn. *Myrica rubra* (Lour.) Siebold & Zucc.) leaves are rich in bioactive compounds but remain underutilized. This study investigated the optimal harvest stage and processing methods to develop high-quality functional powder. We first compared three growth stages: red buds (RB), new leaves (NL), and old leaves (OL). RB exhibited the highest antioxidant capacity and unique volatile profile; however, NL was selected for processing optimization due to the balance between quality and biomass availability. Subsequently, NL was subjected to freeze-drying (FD), mechanical drying (MD), steaming followed by MD (S-MD), and shade drying (SD). Results showed that FD preserved the vibrant green color, glandular trichome structure, ascorbic acid, and fresh volatiles (monoterpenes). Conversely, thermal drying (MD and S-MD) disrupted cellular barriers, which facilitated the extraction of minerals and robust polyphenols like myricitrin, yielding the highest extraction of flavonoids and corresponding antioxidant activity, measured by hydrophilic oxygen radical absorbance capacity (H-ORAC), in hot water extracts than FD. SD significantly degraded quality due to prolonged enzymatic oxidation. While FD is ideal for preserving aesthetics and heat-sensitive nutrients, low-cost MD and S-MD are recommended for producing antioxidant-rich powders for functional food applications where extraction efficiency is prioritized.

## 1. Introduction

Bayberry (*Morella rubra* Lour.; syn. *Myrica rubra* (Lour.) Siebold & Zucc.) is an economically important subtropical fruit tree belonging to the Myricaceae family. It is extensively cultivated in China, with a cultivation area of approximately 334,000 hectares and an annual yield of roughly 950,000 tons [1]. The fruits are rich in antioxidant compounds [1] and are highly valued not only for fresh consumption but also for the production of processed products such as jams, syrups, and juices [2]. Nutritionally, the fruit is drawing attention for its functional properties, reportedly containing high levels of flavonoids and phenolic acids [3]. Numerous studies have demonstrated its health-promoting effects, including antidiabetic [4,5], antioxidant [5], antibacterial and bacteriostatic [6], and anti-ovarian cancer properties [7].

While the fruit has been extensively studied, the health benefits and bioactive constituents of bayberry leaves (BL) have also garnered attention, although to a lesser extent. Zhang, Y. et al. [7] reported that prodelphinidins and proanthocyanidins extracted from the leaves significantly suppressed the proliferation of OVCAR-3 human ovarian cancer cells. Additionally, leaves are rich in myricitrin (MR) and quercetin-3-rhamnoside [8], which exhibit extremely high antioxidant properties [3]. Recent studies have further highlighted the neuropharmacological potential of MR, including antidepressant [9], stress-reducing [10], and anxiolytic effects [11]. Despite the immense potential of BL as a source of bioactive compounds like MR, these biological resources remain largely underutilized. Particularly in Japan, where bayberry trees are commonly planted as street trees, the large biomass of leaves generated during pruning is typically discarded as waste.

To address this issue and add value to this unexploited resource, this study aimed to establish an optimal method for utilizing BL. First, we classified BL into three growth stages: newly sprouted red buds (RB), new leaves (NL), and old leaves (OL). We comprehensively evaluated their health-related functionalities—including antioxidant activities measured by 2,2-diphenyl-1-picrylhydrazyl (DPPH), hydrophilic oxygen radical absorbance capacity (H-ORAC), and 2,2′-azino-bis(3-ethylbenzothiazoline-6-sulfonic acid) (ABTS) assays; total phenolic content (TPC); MR; ascorbic acid; and mineral contents—as well as palatability-related traits such as color and aroma. Furthermore, to identify efficient extraction conditions, the effect of ethanol (EtOH) concentration on the recovery of TPC and MR was examined.

Subsequently, using NL selected for its yield and quality, we investigated processing methods to develop high-quality functional leaf powder. The manufacturing process of tea and herbal powders typically involves thermal treatments that can significantly alter chemical composition and bioactivity [12,13]. Therefore, we examined the effects of steaming treatment and various drying methods—freeze-drying (FD), mechanical drying (MD), and shade drying (SD)—on the retention of polyphenols, antioxidant properties, and volatile components. Although MR is reported to be the primary flavonoid in BL [14], we performed a comprehensive analysis of phenolic constituents using LC-MS/MS and volatile profiles using GC-MS. This study clarifies the relationship between processing conditions (particularly the trade-off between structural preservation and extraction efficiency) and the quality of the final product. Furthermore, given the widespread tradition of consuming plant leaves as herbal teas, the potential application of BL as a tea ingredient was also explored. Hot water extraction was performed to simulate traditional tea brewing conditions, and the extractability of bioactive compounds from the resulting tea infusions was evaluated. The terms “leaf tea”, “powder”, and “tea infusion” are used throughout this manuscript to refer to the dried whole-leaf material after each drying treatment, its pulverized form, and the hot water extract obtained by brewing the powder under simulated tea brewing conditions, respectively.

## 2. Results

### 2.1. Morphological Characteristics and Colorimetric Properties of BL at Different Growth Stages

**Figure 1:**
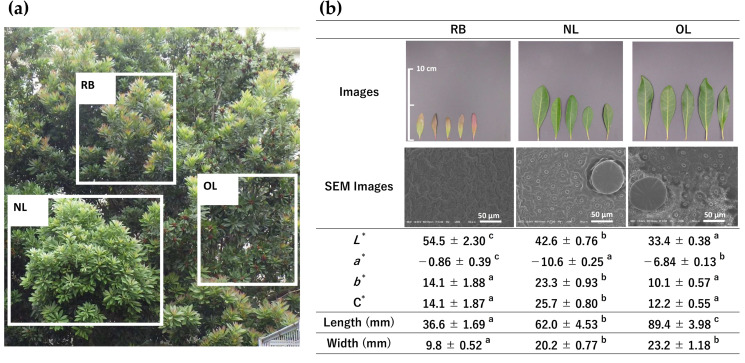

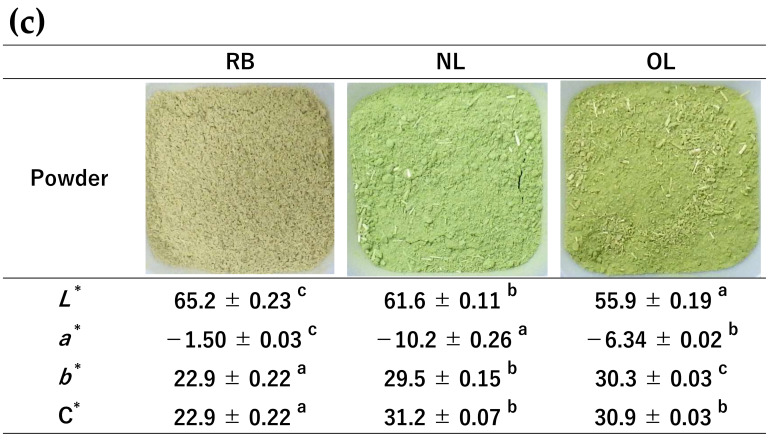
Morphological characteristics of BL at three different growth stages. (**a**) Digital images of fresh leaves (upper row) and processed powders (lower row). (**b**) Scanning electron microscopy (SEM) images of the abaxial leaf surface (scale bar = 50 µm). (**c**) Table summarizing chromatic parameters (L*, a*, b*, and C*), length, and width of fresh leaves and powders. Data are expressed as mean ± SE (*n* = 10 for chromatic parameters *n* = 5 for length and width). Different lowercase letters within the same row indicate significant differences by Tukey’s test (*p* < 0.05). RB, red buds; NL, new leaves; OL, old leaves.

The visual appearance, micromorphology observed by scanning electron microscopy (SEM) and colorimetric parameters of BL and their freeze-dried powders at three growth stages—RB, NL, and OL—are presented in Figure 1.

Bayberry is an evergreen tree, and its leaf morphology undergoes significant changes during maturation. Newly sprouted RB exhibited a reddish hue, whereas NL appeared bright green, transitioning to dark green in OL after more than a year of growth. Leaf dimensions increased significantly with the progression of the growth stage; OL exhibited the greatest length (89.4 ± 3.98 mm) and width (23.2 ± 1.18 mm), followed by NL (62.0 ± 4.53 mm and 20.2 ± 0.77 mm, respectively) and RB (36.6 ± 1.69 mm and 9.8 ± 0.52 mm, respectively).

SEM observations revealed distinct surface microstructures across the different growth stages. In RB, the cellular structure appeared indistinct. In contrast, the more mature stages (NL and OL) exhibited clearly visible stomata and numerous peltate glandular trichomes with a diameter of approximately 70 µm.

Colorimetric analysis quantified these visual differences. For fresh leaves, the lightness (L*) decreased significantly with the progression of the growth stage (RB: 54.5 ± 2.30 > NL: 42.6 ± 0.76 > OL: 33.4 ± 0.38). The redness–greenness coordinate (a*) indicated that NL exhibited the significantly lowest value (−10.6 ± 0.25), representing the strongest green hue, whereas RB displayed the highest value (−0.86 ± 0.39), reflecting its reddish appearance. The yellowness (b*) and chroma (C*) were significantly highest in NL (23.3 ± 0.93 and 25.7 ± 0.80, respectively), followed by RB and OL.

When processed into freeze-dried powder, the color profiles shifted; L*, b*, and C* values increased across all stages compared to fresh leaves. The powder color parameters exhibited distinct trends: L* was highest in RB (65.2 ± 0.23), followed by NL (61.6 ± 0.11) and OL (55.9 ± 0.19). Consistent with fresh leaves, NL powder displayed the lowest a* value (−10.2 ± 0.26) and the highest C* (31.2 ± 0.07), maintaining the most vivid green color among the three stages.

### 2.2. Effect of Extraction Solvent on TPC and MR Content

**Figure 2:**
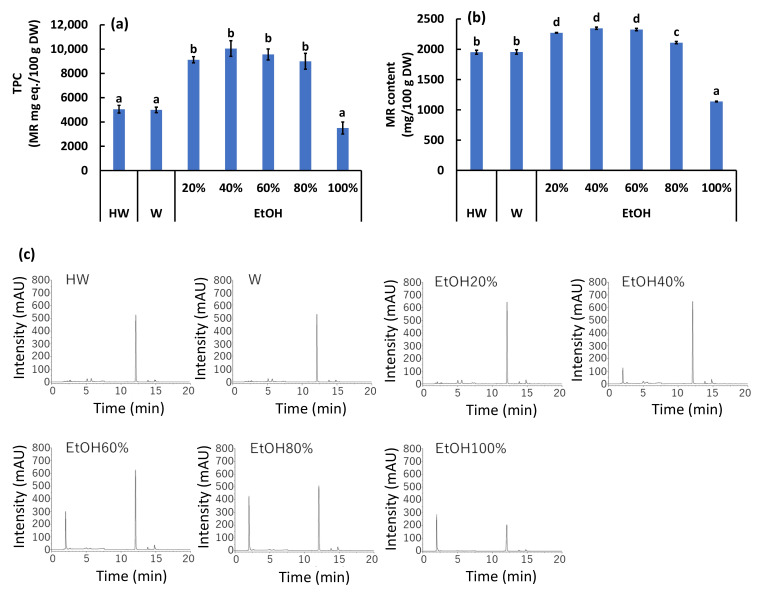
Effect of extraction solvents on total phenolic content (TPC), myricitrin (MR) content, and chromatographic profiles of BL. (**a**) TPC expressed as MR equivalents. (**b**) MR content determined by HPLC. (**c**) HPLC chromatograms of extracts prepared with different solvents: hot water (HW), ambient temperature water (W), and aqueous ethanol solutions (20–100%). MR, myricitrin; HW, hot water (100 °C); W, water (25 °C); EtOH, ethanol; DW, dry weight. Data are expressed as mean ± SE (*n* = 6 for TPC; *n* = 3 for MR content). Different lowercase letters indicate significant differences by Tukey’s test (*p* < 0.05). Note that 60% EtOH was selected as the optimal solvent for subsequent experiments due to its high extraction efficiency and eco-friendly balance.

The extraction efficiency of TPC and MR content from NL varied significantly depending on the EtOH concentration (Figure 2). TPC values were significantly higher (*p* < 0.05) in 20–80% EtOH extracts than in hot water (HW), water (W), and 100% EtOH extracts (Figure 2a). Specifically, 40% EtOH yielded the highest TPC (10,043 ± 639 mg MR eq./100 g DW), which was approximately 2.9-fold higher than the lowest yield obtained with 100% EtOH (3507 ± 492 mg MR eq./100 g DW). Similarly, the MR content was significantly influenced by the solvent composition (Figure 2b). Extracts using 20–60% EtOH showed significantly higher MR levels compared to HW, W, 80%, and 100% EtOH extracts. The 100% EtOH extract resulted in the lowest MR content (1138 ± 7 mg/100 g DW), which was significantly lower than all other extraction conditions. The HPLC chromatograms at 350 nm (Figure 2c) revealed a dominant single peak at a retention time (RT) of approximately 12.0 min, which was subsequently identified as MR by LC-MS/MS analysis. Additionally, a minor peak at RT 2.0 min, presumed to be a disaccharide based on LC-MS/MS data, showed a tendency to increase in intensity with increasing EtOH concentrations up to 80%, whereas it decreased markedly in the 100% EtOH extract.

### 2.3. Variations in Bioactive Compounds, Antioxidant Capacities, and Mineral Profiles Across Leaf Growth Stages

**Figure 3:**
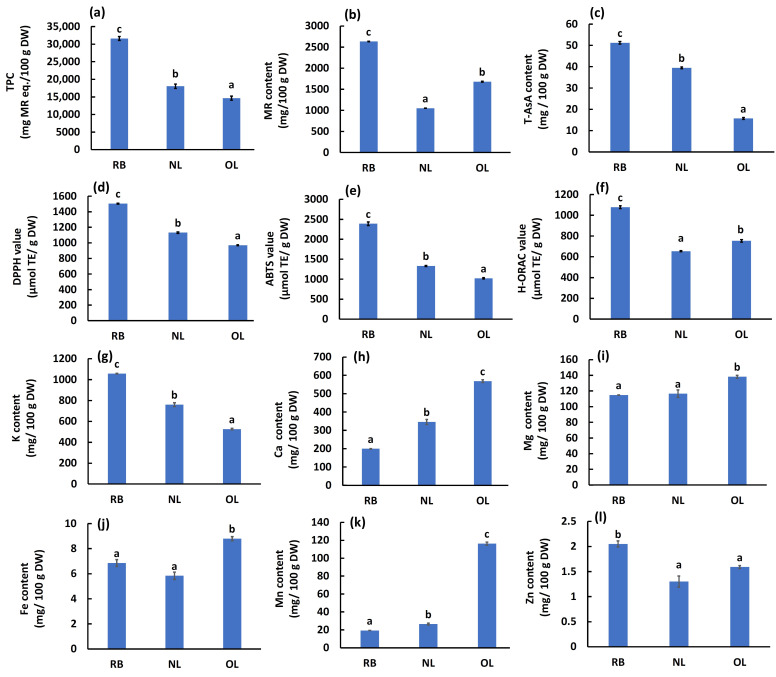
Chemical composition, antioxidant capacities, and mineral profiles of BL at different growth stages. (**a**) Total phenolic content (TPC). (**b**) Myricitrin (MR) content. (**c**) Total ascorbic acid (T-AsA) content. (**d**–**f**) Antioxidant activities determined by DPPH, ABTS, and H-ORAC assays. (**g**–**l**) Mineral contents (K, Ca, Mg, Fe, Mn, Zn). MR, myricitrin; RB, red buds; NL, new leaves; OL, old leaves; DW, dry weight; TE, Trolox equivalents. Data are expressed as mean ± SE (*n* = 6 for TPC, DPPH, and ABTS; *n* = 4 for MR, T-AsA, and H-ORAC; *n* = 3 for minerals). Different lowercase letters indicate significant differences (*p* < 0.05) according to Tukey’s test.

The contents of antioxidant constituents—TPC, MR content, and total ascorbic acid (T-AsA)—varied significantly among the three growth stages: RB, NL, and OL (Figure 3a–c).

TPC and T-AsA content were highest in RB (31,600 ± 560 mg MR eq./100 g DW and 51.2 ± 0.6 mg/100 g DW, respectively), followed by NL and OL, showing a decreasing trend with leaf maturation. MR content peaked in RB (2631 ± 7.3 mg/100 g DW) and was lowest in NL (1051 ± 2.5 mg/100 g DW), with OL exhibiting an intermediate value (1678 ± 11 mg/100 g DW). The proportion of MR relative to TPC was calculated as 8.3%, 5.8%, and 11.5% in RB, NL, and OL, respectively.

Antioxidant activities evaluated by three assays showed distinct trends (Figure 3d–f). DPPH and ABTS radical scavenging activities followed the order: RB > NL > OL, consistent with the trends in TPC and T-AsA. Specifically, RB exhibited the highest activity in both assays. In contrast, H-ORAC values showed a different pattern: RB > OL > NL, which closely mirrored the trend observed for MR content.

The mineral composition (K, Ca, Mg, Fe, Mn, Zn) changed dynamically with growth (Figure 3g–l). Potassium (K) contents (mg/100 g DW) significantly decreased as the leaves matured (RB > NL > OL), whereas zinc (Zn) was highest in RB with no significant difference observed between NL and OL. Conversely, calcium (Ca), magnesium (Mg), iron (Fe), and manganese (Mn) contents increased with leaf growth, with OL exhibiting the significantly highest concentrations for these minerals.

### 2.4. LC-MS/MS Profiling of Phenolic Compounds in BL

**Figure 4:**
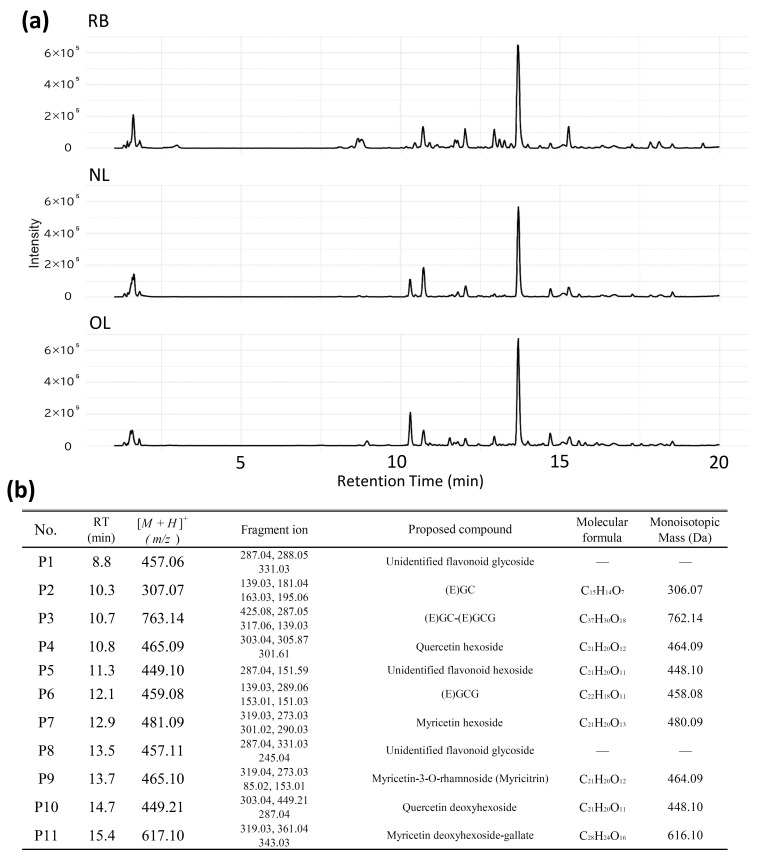
LC-MS/MS analysis of phenolic compounds in BL. (**a**) Base peak chromatograms (BPCs) of 60% ethanol extracts from red buds (RB), new leaves (NL), and old leaves (OL). (**b**) List of detected peaks including their retention times (RT), precursor ions ([*M* + *H*]^+^), characteristic fragment ions, and proposed compounds. The peak numbers (P1–P11) correspond to the retention times observed in the chromatograms. (E)GC: (epi)gallocatechin, (E)GCG: (epi)gallocatechin gallate.

The base peak chromatograms (BPCs) obtained from the LC-MS/MS analysis of 60% EtOH extracts of RB, NL, and OL are shown in Figure 4. A dominant peak was observed at a RT of 13.7 min in all samples. Furthermore, RB, which exhibited the highest antioxidant activity, displayed a more complex profile with several minor peaks that were not detected in NL or OL. The BPCs of NL and OL showed similar chromatographic patterns.

Based on the MS/MS spectral analysis, the detected peaks were classified into four groups according to their characteristic fragment ions. First, peaks yielding a fragment ion at *m*/*z* 287 included P1 (RT 8.8 min, [*M* + *H*]^+^ *m*/*z* 457.06), P5 (RT 11.3 min, *m*/*z* 449.10), and P8 (RT 13.5 min, *m*/*z* 457.11). Second, a fragment ion at *m*/*z* 139 was characteristic of P2 (RT 10.3 min, *m*/*z* 307.07), P3 (RT 10.7 min, *m*/*z* 763.14), and P6 (RT 12.1 min, *m*/*z* 459.08). Third, peaks P4 (RT 10.8 min, *m*/*z* 465.09) and P10 (RT 14.7 min, *m*/*z* 449.21) produced a fragment ion at *m*/*z* 303. Finally, the fragment ion at *m*/*z* 319 was observed in P7 (RT 12.9 min, *m*/*z* 481.09), P9 (RT 13.7 min, *m*/*z* 465.10), and P11 (RT 15.4 min, *m*/*z* 617.10).

### 2.5. GC-MS Profiling of Volatile Compounds Across Growth Stages

**Figure 5:**
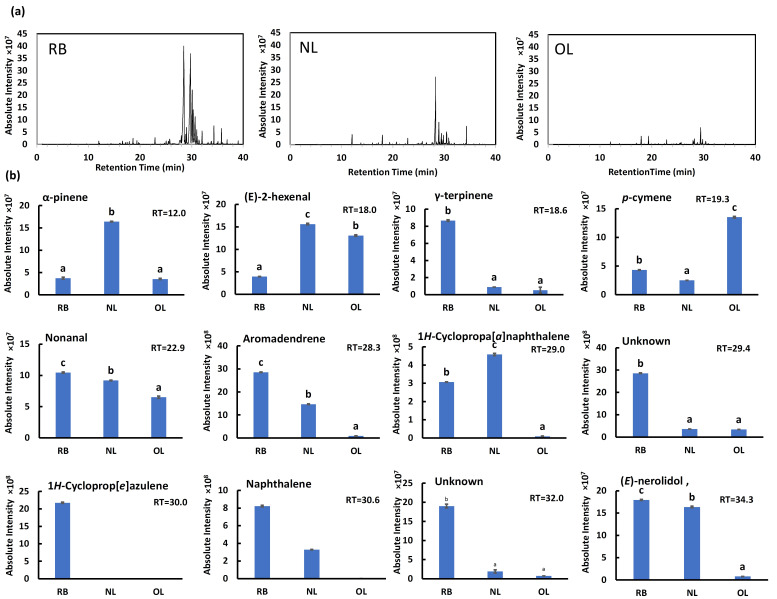
GC-MS profiles of volatile compounds in BL at different growth stages. (**a**) Total ion chromatograms (TICs) of volatile compounds from leaf powder of red buds (RB), new leaves (NL), and old leaves (OL). (**b**) Relative abundance (absolute intensity) of representative volatile compounds identified in the samples. RB, red buds; NL, new leaves; OL, old leaves. Data are expressed as mean ± SE (*n* = 3). Different lowercase letters indicate significant differences (*p* < 0.05) according to Tukey’s test.

The total ion chromatograms (TICs) for the three growth stages—RB, NL, and OL—are shown in Figure 5a. While numerous peaks were detected across all samples, the RB profile was distinctively characterized by a cluster of high-intensity peaks around the retention time (RT) of 30 min. The total peak areas for RB, NL, and OL were calculated as 1.20 × 10^10^, 4.10 × 10^9^, and 1.64 × 10^9^, respectively. These results indicate that RB contained a significantly higher total abundance of volatile compounds compared to the other stages.

The identified compounds showed distinct accumulation patterns depending on the growth stage (Figure 5b). Several compounds were dominant in RB, significantly decreasing with maturation (RB > NL > OL). Specifically, γ-terpinene was approximately 9.7-fold and 16.2-fold higher in RB than in NL and OL, respectively. Other compounds following this trend included aromadendrene, 1*H*-cycloprop[*e*]azulene, naphthalene, an unknown compound, and (*E*)-nerolidol. Notably, 1*H*-cycloprop[*e*]azulene was exclusively detected in RB with an extremely high peak area (2.17 × 10^9^) and was absent in the later stages.

In contrast, specific volatile compounds were most abundant in NL. These included α-pinene, (*E*)-2-hexenal, and 1*H*-cyclopropa[*a*]naphthalene, which peaked at the new leaf stage. Conversely, *p*-cymene exhibited a unique accumulation pattern distinct from other volatiles, being markedly highest in OL.

Regarding other detected components, nonanal was present across all growth stages but maintained higher levels in RB and NL compared to OL. Additionally, RT 29.4 min and RT 32.0 min, although unidentified, were noted as characteristic peaks in the RB chromatogram.

### 2.6. Effect of Drying Methods on the Appearance and Microstructure of NL

**Figure 6:**
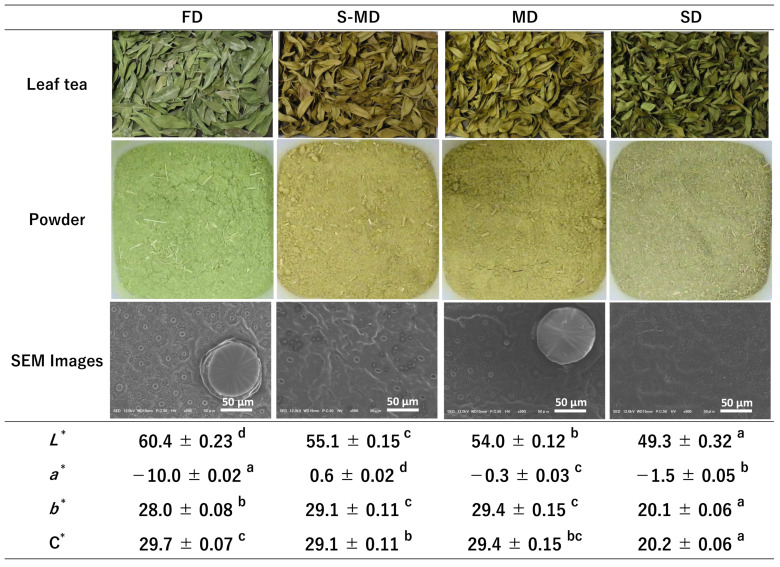
Impact of different drying methods on the appearance and microstructure of new leaves (NL). Digital images of leaf tea (upper row) and processed powder (lower row). Scanning electron microscopy (SEM) images of the abaxial leaf surface showing peltate glandular trichomes (scale bar = 50 µm). Table of chromatic parameters (L*, a*, b*, and C*) for the processed powders. FD, freeze-drying; S-MD, steaming followed by mechanical drying; MD, mechanical drying; SD, shade drying. Data are expressed as mean ± SE (*n* = 5). Different lowercase letters within the same row indicate significant differences by Tukey’s test (*p* < 0.05).

The visual appearance of the processed leaves (hereafter referred to as “leaf tea”, denoting the dried whole-leaf material prior to pulverization) and processed powder prepared by four different drying methods—FD, steaming followed by mechanical drying (S-MD), MD, and SD—is shown in Figure 6.

The FD samples retained a vibrant green color in both leaf and powder forms, comparable to fresh leaves. Quantitative colorimetric measurements performed on the leaf powders corroborated this observation; the FD powder exhibited the significantly highest lightness (L* = 60.4 ± 0.23) and the lowest a* value (a* = −10.0 ± 0.02), indicating the strongest green hue.

In contrast, powders derived from thermal drying methods (S-MD and MD) and SD exhibited marked discoloration. The S-MD and MD powders appeared yellowish-brown, with a* values shifting to 0.6 ± 0.02 and −0.3 ± 0.03, respectively. The SD powder yielded the darkest appearance, exhibiting the significantly lowest L* value of 49.3 ± 0.32.

SEM imaging of the leaf surface revealed distinct morphological differences in the peltate glandular trichomes among the drying treatments (Figure 6).

The FD process preserved the cellular microstructure exceptionally well; the peltate glandular trichomes remained intact and spherical with a smooth surface, showing no signs of collapse or rupture. Conversely, the trichomes in S-MD samples were severely collapsed and flattened. MD samples retained a relatively spherical shape but appeared slightly deformed compared to FD. In SD samples, the trichomes were shrunken and structurally indistinct.

### 2.7. Effect of Drying Methods on Chemical Composition, Antioxidant Activities, and Mineral Content

**Figure 7:**
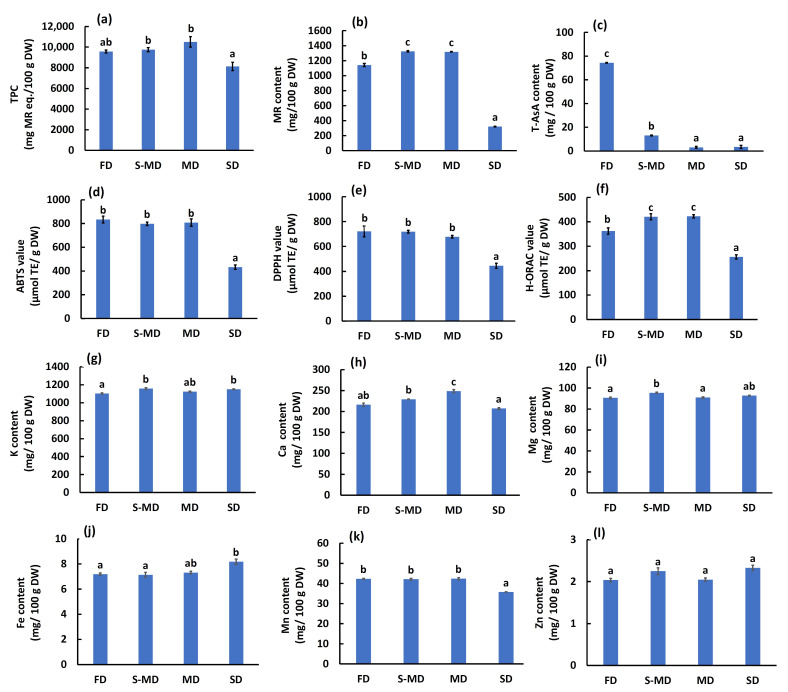
Effect of different drying methods on chemical composition, antioxidant activities, and mineral contents of new leaves (NL). (**a**) Total phenolic content (TPC). (**b**) Myricitrin (MR) content. (**c**) Total ascorbic acid (T-AsA) content. (**d**–**f**) Antioxidant activities determined by ABTS, DPPH, and H-ORAC assays, respectively. (**g**–**l**) Mineral contents (K, Ca, Mg, Fe, Mn, Zn). FD, freeze-drying; S-MD, steaming followed by mechanical drying; MD, mechanical drying; SD, shade drying. Data are expressed as mean ± SE (*n* = 6 for TPC, ABTS, and DPPH; *n* = 4 for MR, T-AsA, and H-ORAC; *n* = 3 for minerals). Different lowercase letters indicate significant differences (*p* < 0.05) according to Tukey’s test.

The quantitative analysis of TPC, specific bioactive compounds (MR and T-AsA), antioxidant activities, and mineral contents revealed significant differences among the drying methods (Figure 7). The MR content was significantly higher in thermal drying methods, specifically S-MD and MD (1325 ± 8.9 and 1318 ± 4.1 mg/100 g DW, respectively) compared to FD (1142 ± 21 mg/100 g DW) and SD (322 ± 4.5 mg/100 g DW) (Figure 7b). A similar trend was observed for TPC, where MD and S-MD samples exhibited slightly higher values than FD (Figure 7a). In stark contrast, T-AsA was best preserved in FD samples (74.4 ± 0.3 mg/100 g DW) (Figure 7c). S-MD samples showed a drastic reduction in T-AsA (13.1 ± 0.4 mg/100 g DW), while MD and SD samples exhibited almost complete depletion. The antioxidant activities showed assay-dependent trends (Figure 7d–f). For the H-ORAC assay, S-MD and MD samples exhibited significantly higher activity (420 ± 12 and 422 ± 6.7 µmol TE/g DW, respectively) compared to FD (362 ± 13 µmol TE/g DW) (Figure 7f). However, for ABTS and DPPH assays, FD showed activities comparable to or slightly higher than thermal drying methods. SD consistently showed the lowest antioxidant activity across all assays.

Regarding mineral content (K, Ca, Mg, Fe, Mn, Zn), the trends varied by element (Figure 7g–l). For Ca, the MD treatment resulted in the significantly high content (Figure 7h), while Mg levels were significantly highest in S-MD samples (Figure 7i). Interestingly, Fe content showed a unique pattern, being significantly highest in SD samples (Figure 7j). Zn levels showed no significant differences among the drying methods (Figure 7l).

### 2.8. Effect of Drying Methods on Volatile Profiles

**Figure 8:**
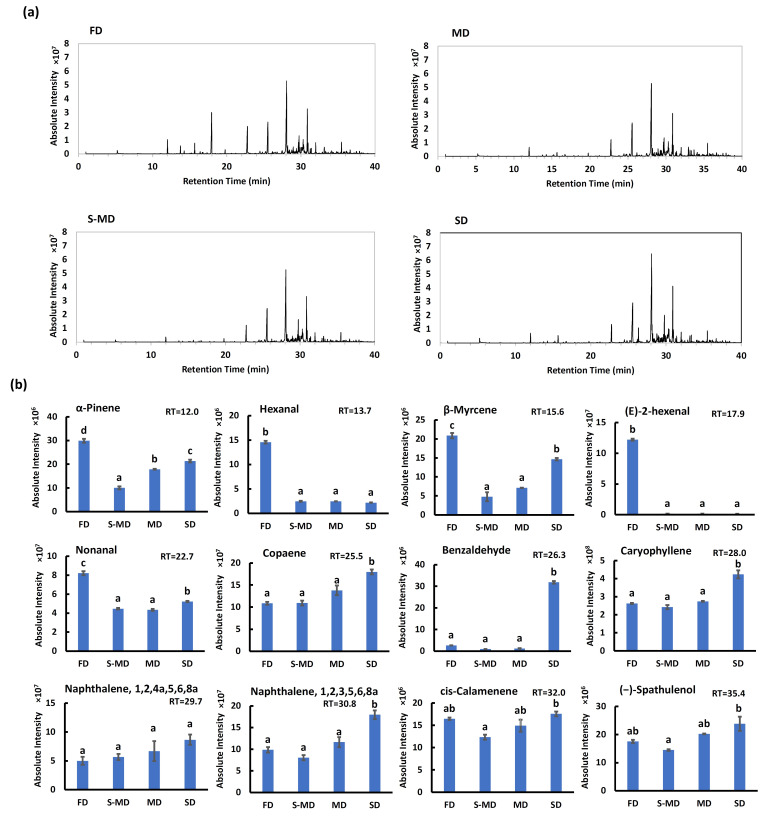
Effect of drying methods on the volatile profiles of new leaves (NL). (**a**) Total ion chromatograms (TICs) of volatile extracts from hot water infusions (tea infusions; prepared by extracting leaf powder with boiling water to simulate traditional tea brewing, as described in Section 4.4.3) of freeze-drying (FD), mechanical drying (MD), steaming followed by mechanical drying (S-MD), and shade drying (SD) leaf powders. (**b**) Relative abundance (absolute intensity) of representative volatile compounds. FD, freeze-drying; S-MD, steaming followed by mechanical drying; MD, mechanical drying; SD, shade drying. Data are expressed as mean ± SE (*n* = 3). Different lowercase letters indicate significant differences (*p* < 0.05) according to Tukey’s test.

The GC-MS analysis identified distinct volatile profiles among the drying treatments (Figure 8).

Freeze-dried (FD) samples were characterized by the highest retention of monoterpenes and aliphatic aldehydes, which are responsible for fresh and green aromas. Specifically, the peak areas of α-pinene, β-myrcene, hexanal, (*E*)-2-hexenal, and nonanal were significantly higher in FD samples (Figure 8b).

In contrast, the thermal drying methods (S-MD and MD) resulted in a substantial loss of these volatile compounds. Notably, S-MD samples exhibited the lowest content across most volatile classes, with almost negligible amounts of hexanal and (*E*)-2-hexenal.

SD produced a unique profile characterized by the accumulation of specific compounds. Most remarkably, benzaldehyde was detected at an extremely high level exclusively in SD samples, being nearly absent or very low in other treatments (Figure 8b). Furthermore, SD samples contained the significantly highest levels of sesquiterpenes, including caryophyllene, copaene, and (−)-spathulenol, as well as naphthalene derivatives.

## 3. Discussion

### 3.1. Evaluation of Leaf Quality Based on Appearance and Color

The choice of leaf growth stage is a critical factor determining both the yield and the visual quality of the final product. The morphological data indicated that OL provides the highest biomass yield due to its significantly larger leaf size, suggesting its suitability for applications where production efficiency is the primary objective.

However, for food applications where visual appeal is paramount, color quality is crucial. Although OL offered a yield advantage, its processed powder exhibited a lower L* value (darker appearance) and reduced C* compared to NL. In contrast, NL powder demonstrated the strongest green hue (most negative a* value) and high saturation, characterized by a vivid bright green color. Therefore, despite the lower individual leaf mass compared to OL, NL is considered the most suitable stage for producing high-quality functional powders, particularly for use as a natural green colorant or tea ingredient.

The SEM observations of peltate glandular trichomes in NL and OL are consistent with the morphology of “sunken trichomes” (flat, round structures) reported in the related species *Myrica pensylvanica* [15]. The clarity of these structures in NL and OL, compared to the distinct immature morphology in RB, suggests that the secretory tissues are fully developed in these stages. This structural difference may influence the retention of volatile components and bioactive compounds, as discussed in later sections.

### 3.2. Optimization of Extraction Solvents for Maximizing Bioactive Compound Recovery

The polarity of the extraction solvent plays a pivotal role in the recovery of bioactive polyphenols. Our findings indicate that binary solvent systems of EtOH and water (specifically 20–60% EtOH) are superior to mono-solvent systems (pure water or pure EtOH) for extracting TPC and MR from BL. This trend aligns with previous studies on other plant matrices; for example, 50% EtOH was reported to exhibit the highest antioxidant activity in yerba mate [16], and 40–80% EtOH solutions were optimal for other medicinal plants [17,18].

The poor extraction efficiency observed with 100% EtOH can be attributed to the chemical nature of the target compounds. MR is a flavonoid glycoside (myricitrin; myricetin-3-*O*-rhamnoside), which possesses higher polarity than its aglycone due to the sugar moiety [8,19]. Therefore, the presence of water in the solvent matrix is essential to enhance the solubility and mass transfer of these glycosylated phenolics from the plant tissue [20,21]. Although 40% EtOH yielded the numerically highest TPC, no statistical difference was observed between 40% and 60% EtOH (Figure 2a). Therefore, 60% EtOH was selected as the optimal solvent for the subsequent comprehensive evaluation, considering the balance between high extraction efficiency and the ease of solvent removal (evaporation energy) for industrial applications [22].

### 3.3. Nutritional Assessment and Selection of Optimal Harvest Stage Based on Functional Components of BL

The metabolic profile of BL changes drastically during development, offering different functional benefits depending on the harvest stage. RB accumulated the highest levels of antioxidant compounds, particularly TPC and T-AsA, resulting in superior DPPH and ABTS radical scavenging activities. This high antioxidant capacity in young tissues is likely a defense mechanism against oxidative stress during rapid growth. However, the relatively low proportion of MR in TPC (approx. 6–11%) suggests the presence of other significant phenolic compounds. Previous studies have reported myricetin glycosides and quercetin glycosides [19] as well as proanthocyanidins [23,24] in the leaves, implying that RB may contain high concentrations of these non-MR polyphenols.

The discrepancy observed in the H-ORAC assay (where OL > NL) compared to DPPH/ABTS (where NL > OL) can be attributed to the reaction mechanisms. DPPH and ABTS assays are primarily based on the Single Electron Transfer (SET) mechanism, whereas the H-ORAC assay is based on the Hydrogen Atom Transfer (HAT) mechanism [25]. Since H-ORAC utilizes biologically relevant peroxyl radicals, it may better reflect the radical chain-breaking capacity of specific antioxidants like MR, which followed the same trend (RB > OL > NL). This suggests that MR plays a dominant role in the H-ORAC activity of BL.

Regarding mineral nutrition, the distinct accumulation patterns observed allow for targeted harvesting based on specific dietary requirements. RB is identified as the optimal source for Zn and K supplementation. Since Zn deficiency is closely linked to taste disorders and immune function, and K promotes sodium excretion, RB-derived products could be targeted towards these specific health needs. In contrast, OL serves as a superior source of Ca, Mg, Fe and Mn. Given that Ca and Mg are vital for bone health, and Mn acts as a cofactor for numerous metabolic enzymes [26], OL—which is typically discarded during pruning—can be effectively valorized as a mineral-rich ingredient. Therefore, producers can strategically select the harvest stage: RB for antioxidant and Zn-focused products, and OL for broad-spectrum mineral fortification, thereby maximizing the utility of the biological resource.

### 3.4. Identification and Metabolic Changes in Phenolic Compounds During Leaf Maturation

#### 3.4.1. Myricetin Derivatives

The fragment ion at *m*/*z* 319, observed using the base peaks of P9, P7, and P11 as precursor ions, corresponds to myricetin aglycone (318 Da) [27] based on existing literature. For P9, the fragment ion is consistent with the cleavage pattern of myricetin 3-*O*-rhamnoside reported by Yang et al. [19]. Furthermore, the RT coincided with the maximum peak in the UV chromatogram at 254 nm and was consistent with that of the MR standard measured in Section 3.3. Consequently, these results strongly suggest that P9 is MR [28,29]. For P11, the observed neutral loss is consistent with the sequential loss of a rhamnose residue (146 Da) [30] and a galloyl group (152 Da) [31]; thus, it is considered to be myricetin deoxyhexoside-gallate. For P7, the neutral loss corresponds to a hexose residue (162 Da) [30], identifying it as myricetin hexoside.

#### 3.4.2. Catechin Derivatives

In the MS/MS analysis of P2 and P6, a fragment ion at *m*/*z* 139 was detected. This is interpreted as a characteristic ion resulting from Retro-Diels-Alder (RDA) cleavage of the catechin skeleton. The mass of the precursor ion in P2 (*m*/*z* 307) and the characteristic peak at *m*/*z* 139 match the theoretical values for (epi)gallocatechin ((E)GC) (MW 306) [29], suggesting that this component is (E)GC. In P6, a fragment ion at *m*/*z* 289.06 was observed in addition to *m*/*z* 139, which corresponds to the loss of a gallic acid moiety (170 Da) [30]. Based on this fragmentation pattern indicating the presence of a galloyl group, this component is presumed to be (epi)gallocatechin gallate ((E)GCG, MW 458) [24,32,33]. P3 is consistent with the theoretical value (MW 762) [24,29] for a dimer formed by the condensation of an (epi)gallocatechin monomer (306 Da) and an (E)GCG monomer (458 Da) [24,33] via the loss of two hydrogen atoms (i.e., (E)GC-(E)GCG). The fragment ion at *m*/*z* 425.08 [24,29] indicates bond cleavage between the flavan skeletons and characteristic decomposition related to the galloyl group [24].

#### 3.4.3. Quercetin Derivatives

The *m*/*z* 303 fragment ion observed in P4 and P10 is attributed to quercetin aglycone (302 Da) [32] formed by the neutral loss of a sugar moiety. P4 is suggested to be a quercetin glycoside (hexose) resulting from the neutral loss of a hexose residue (162 Da) [30], and P10 is suggested to be a quercetin glycoside (rhamnose) resulting from the loss of a rhamnose residue (146 Da) [30].

#### 3.4.4. Presumed Kaempferol Derivatives

The fragment ion *m*/*z* 287 observed in the MS/MS analysis of P1, P5, and P8 is presumed to correspond to the aglycone of kaempferol [32,34] or luteolin [35] (MW 286). P5 is thought to arise from the elimination of a hexose residue (162 Da) [30] from the precursor ion, and previously reported kaempferol glycosides are possible candidates. P1 and P8 are presumed to be kaempferol or luteolin glycosides, although such compounds have not been previously reported in bayberry or related species. Notably, both P1 and P8 were specific to RB; in contrast, no clear peaks were detected in NL or OL. Kaempferol derivatives are widely recognized for their significant pharmacological potential, particularly exhibiting strong antioxidant and anticancer activities [36]. Consequently, the unique peaks P1 and P8, which were identified exclusively in the RB sample, warrant further investigation to elucidate their specific structural characteristics and functional contributions. Although the specific compounds could not be definitively identified in this study, the results suggest they are components lost during maturation and may not have been analyzed in previous studies focusing on mature leaves.

### 3.5. Dynamic Changes in Volatile Profiles and Mechanisms of Accumulation Without Glandular Trichomes

#### 3.5.1. Reported Biological Activities of Identified Volatile Compounds

Several of the identified volatile compounds possess previously reported biological activities relevant to food applications. α-pinene and γ-terpinene, abundant in NL and RB respectively, are known for their anti-inflammatory and antioxidant properties [37,38,39], while (*E*)-2-hexenal exhibits antimicrobial activity [40]. *p*-cymene, enriched in OL, possesses diverse pharmacological activities including antioxidant and antimicrobial effects [41]. Additionally, nonanal and (*E*)-nerolidol are reported to have antifungal and broad-spectrum antimicrobial properties, respectively [42,43]. The stage-dependent accumulation of these bioactive compounds suggests that targeted harvesting could be employed to optimize BL for specific functional applications; for instance, RB for antioxidant-rich extracts and OL for antimicrobial applications.

#### 3.5.2. Biosynthetic Mechanisms in Early Development

A contradiction was observed regarding the storage sites of these volatiles. SEM observations (Figure 1) revealed that RB leaves were undeveloped and lacked peltate glandular trichomes, which were clearly observed on the surface of NL and OL. Since glandular trichomes are generally considered the primary storage sites for essential oils [44], it was initially hypothesized that RB would contain lower levels of volatiles. However, the GC-MS analysis revealed the opposite: RB contained the highest total amount of volatiles. This phenomenon suggests the existence of alternative biosynthetic or storage mechanisms during the early stages of leaf development.

First, the high concentration of nonanal in RB is likely linked to the dynamic metabolism of the cuticle layer during rapid leaf expansion. It has been demonstrated that cuticular wax components, specifically alkenes, undergo spontaneous oxidative cleavage to generate volatile aldehydes, including nonanal [45]. Therefore, nonanal in RB may originate from the breakdown of wax precursors during cuticle formation rather than from glandular secretion.

Second, the dominance of sesquiterpenes (e.g., aromadendrene and 1*H*-cycloprop[*e*]azulene) suggests that internal tissues may function as storage sites in young leaves. Previous studies have identified “internal glands” within the mesophyll of other species (*Pogostemon cablin*), which serve as sites for terpene biosynthesis independent of surface trichomes [46]. It is plausible that BL also possess intracellular compartments (e.g., vacuoles or lipid bodies) for volatile storage in their undifferentiated tissues [47].

Finally, this phenomenon can be interpreted as a defense trade-off. Since RB leaves lack physical barriers such as a thickened cuticle or mature trichomes, they may prioritize chemical defense. Immature leaves have been proposed to function as dominant “volatile sensing organs” with heightened metabolic responsiveness [48]. The significantly higher levels of γ-terpinene in RB likely serve as a chemical shield against oxidative stress and herbivory before physical defenses are fully established.

### 3.6. Impact of Drying Processes on Tissue Integrity and Quality Attributes

The choice of drying method critically influenced the structural integrity and color quality of bayberry leaf powders. FD was identified as the superior method for preserving the original tissue architecture and pigmentation [49]. Unlike thermal drying (S-MD, MD), which induces chlorophyll degradation via conversion to pheophytins or pheophorbides [50], or SD which facilitates enzymatic oxidation due to the prolonged drying period and lack of heat inactivation [51], FD dehydrates the tissue via sublimation, effectively arresting degradative reactions and resulting in a bright green powder.

Furthermore, the intact state of the peltate glandular trichomes in FD samples confirmed that the physical barriers of the leaf tissue—including the cuticle layer and cell membranes—remained unbroken [52,53]. In contrast, the cellular collapse observed in S-MD samples indicates disruption of cell walls and membranes caused by steaming and thermal stress. This difference in structural integrity has critical implications for both volatile retention and the extractability of bioactive compounds, as discussed in the following sections.

### 3.7. The Trade-Off Between Compound Stability and Extractability in Drying Processes

T-AsA is highly thermolabile and susceptible to oxidation. The superior retention of T-AsA in FD samples confirms that low-temperature sublimation is a highly effective method for preserving heat-sensitive vitamins [49], while the drastic loss observed in S-MD, MD samples is attributed to thermal degradation, and the near-complete depletion in SD samples is likely due to prolonged enzymatic oxidation [51].

These compositional changes directly influenced the antioxidant evaluations. The high T-AsA content in FD samples likely compensated for lower phenolic extractability, resulting in DPPH and ABTS activities comparable to those of thermally dried samples. Conversely, the higher MR content and H-ORAC values in S-MD and MD samples strongly support the trade-off between structural integrity and extraction efficiency. As indicated by the SEM observations (Figure 6), the intact cellular structure of FD-treated BL paradoxically restricts solvent penetration and mass transfer of intracellular metabolites [54]. It should be noted that the Folin–Ciocalteu reagent also reacts with ascorbic acid [25]; the comparable TPC values between FD and S-MD therefore imply that the actual phenolic extractability was superior in thermally processed samples. Thermal treatments disrupt cell membranes and the cell wall matrix, enhancing the extractability of bound phenolics such as MR and facilitating the release of cell wall-bound minerals [12,50].

### 3.8. Mechanisms of Volatile Retention and Enzymatic Transformation During Drying

The drying process profoundly influenced the retention and transformation of volatile compounds, highlighting a critical trade-off between structural preservation and component volatilization.

The superior retention of high-volatility compounds (e.g., monoterpenes and aldehydes) in FD samples can be directly attributed to the preservation of peltate glandular trichomes, as observed in the SEM images (Figure 6). Since these trichomes serve as the primary storage sites for essential oils [52], keeping them intact under low-temperature vacuum conditions effectively prevents the volatilization of these stored compounds. This finding aligns with previous reports indicating that FD maximizes the retention of terpenes in essential oil-bearing plants [55]. The abundance of (*E*)-2-hexenal (a green leaf volatile) in FD samples further confirms that the characteristic fresh aroma profile was successfully preserved without thermal degradation.

Conversely, the S-MD treatment resulted in the poorest volatile profile, which stands in sharp contrast to its high phenolic extractability (Figure 7). This divergence is explained by the physical state of the tissue; the rupture of trichomes and cell membranes caused by steaming (Figure 6), while beneficial for extracting non-volatile phenolics, proved highly detrimental to the retention of volatiles. The disruption of these storage structures exposed the essential oils to heat, causing them to evaporate or degrade during the subsequent drying process. Indeed, thermal processing has been reported to cause a significant decrease in volatile alcohols and esters in tea leaves [13]. This demonstrates that structural disruption promotes the loss of volatiles, whereas it enhances the extraction of non-volatile components.

The unique profile of SD samples, particularly the marked accumulation of benzaldehyde, indicates the occurrence of active metabolic reactions during the prolonged drying period. Benzaldehyde is often formed through the enzymatic oxidation of precursor compounds such as L-phenylalanine via phenylpyruvic acid [56] or the degradation of glycosides (e.g., prunasin or amygdalin) [57]. Since SD does not immediately inactivate endogenous enzymes—unlike thermal or freezing methods— it allowed these enzymatic conversions to proceed, resulting in a distinct, oxidized aroma profile. The higher relative abundance of sesquiterpenes in SD might also result from a concentration effect as lighter monoterpenes evaporated over time, or potentially due to stress-induced biosynthesis during the slow dehydration process.

## 4. Materials and Methods

### 4.1. Plant Materials

Bayberry leaves were collected from trees located on the campus of Shimane University (Matsue, Shimane, Japan) in June 2023. To investigate the potential valorization of agricultural waste, leaf samples were obtained from branches discarded during routine pruning. The harvested leaves were manually classified into three distinct growth stages based on their morphological characteristics and maturity: RB, which are immature, unexpanded leaves characterized by reddish pigmentation; NL, representing fully expanded green leaves originating from the current year’s growth; and OL, which are mature, dark green leaves from the previous year’s growth (overwintered). Representative digital images of these materials, along with their morphological parameters (color, length, and width), are presented in Figure 1. All samples were washed with distilled water to remove dust and surface impurities prior to processing.

### 4.2. Color Measurement

The chromatic properties of the leaf surfaces were evaluated using a tristimulus colorimeter (CR-13, Konica Minolta, Osaka, Japan). The CIELAB color space coordinates, including lightness (L*), redness-greenness (a*), and yellowness-blueness (b*), were recorded. Additionally, chroma (C*), representing color saturation, was calculated using the following Equation (1):

(1) $$C*\;=\sqrt{a^{*2}+b^{*2}}$$

Ten replicates were measured per sample.

### 4.3. SEM

The surface micromorphology of the leaf samples was visualized using SEM. To assess the structural integrity of the glandular trichomes, the abaxial surfaces of both the freeze-dried raw materials (RB, NL, and OL) and the processed leaf teas were examined. Small leaf sections (approximately 3 × 3 mm) were excised and mounted onto aluminum stubs (Type-HM, Nissin EM Corporation, Tokyo, Japan) using conductive double-sided carbon tape (Nissin EM Corporation). Subsequently, the specimens were sputter-coated with a thin layer of gold to prevent charging. Imaging was performed using a JSM-IT100 scanning electron microscope (JEOL Ltd., Tokyo, Japan) at an accelerating voltage of 10 kV, with representative images captured at 500× magnification.

### 4.4. Preparation of Leaf Extracts

#### 4.4.1. Optimization of Extraction Efficiency Based on Solvent Composition

To determine the optimal extraction conditions, the effect of EtOH concentration on the recovery of total phenolics and MR from NL was evaluated. EtOH was selected as the extraction solvent because it is a food-grade solvent widely accepted for use in food and functional food applications, offers favorable miscibility with water to form binary solvent systems that enhance the extractability of polyphenols, and is readily removed after extraction due to its relatively low boiling point [16,17,18]. A series of aqueous EtOH solutions with varying concentrations (0, 20, 40, 60, 80, and 100% *v*/*v*) was tested. Briefly, 200 mg of pulverized leaf powder was suspended in 10 mL of each solvent and incubated under shaking conditions at 60 °C for 2 h. In addition, hot water extraction was performed to simulate traditional tea brewing conditions, given the potential application of BL as a tea ingredient (as described in the Section 1). Leaf powder (200 mg) was added to 20 mL of distilled water and boiled at 100 °C for 10 min. After cooling, the mixture was adjusted to a final volume of 50 mL with distilled water. All resulting extracts were filtered through a 0.45 µm membrane filter prior to analysis.

#### 4.4.2. Comparative Analysis of Antioxidant Components and Properties Across Growth Stages

To evaluate the impact of leaf maturity on chemical profiles and bioactivity, extracts were prepared from the three raw materials (RB, NL, and OL) using the optimized solvent condition determined in Section 4.4.1 (60% EtOH). The extraction process was identical to that described above. The resulting extracts were subjected to a comprehensive suite of analyses, including the determination of TPC, evaluation of antioxidant capacity via 2,2-diphenyl-1-picrylhydrazyl (DPPH) radical scavenging and hydrophilic oxygen radical absorbance capacity (H-ORAC) assays, and phytochemical profiling using high-performance liquid chromatography (HPLC) and ultra-high-performance liquid chromatography–electrospray ionization tandem mass spectrometry (UHPLC-ESI-MS/MS).

#### 4.4.3. Evaluation of Processed Leaf Teas

To evaluate the quality of the processed leaves (FD, S-MD, MD, and SD) under realistic consumption conditions, hot water extraction was employed to simulate traditional tea brewing. The extraction procedure was identical to the hot water method described in Section 4.4.1. Specifically, the processed leaf powders were extracted with boiling water, and the resulting infusions were analyzed to determine the retention and extractability of bioactive compounds and their corresponding antioxidant activities.

### 4.5. Quantitative Analysis of MR via HPLC

The quantification of MR was performed using a Hitachi LaChrom HPLC system (Hitachi Ltd., Tokyo, Japan). Chromatographic separation was achieved on an InertSustain Swift C18 column (4.6 × 150 mm; GL Sciences Inc., Tokyo, Japan) maintained at 40 °C. The mobile phase consisted of (A) 0.1% formic acid in water and (B) 0.1% formic acid in acetonitrile, delivered at a flow rate of 1.0 mL/min. The gradient elution program was set as follows: initial hold at 10% B (90:10) for 0–2 min; linear increase to 35% B (65:35) over the next 13 min (2–15 min); isocratic hold at 35% B for 5 min (15–20 min); and a rapid increase to 95% B (5:95) by 20.1 min. UV detection wavelengths were switched during the run: 280 nm was used for the period 0–7.5 min, followed by 370 nm for 7.5–60 min. An authentic standard of MR was obtained from Tokyo Chemical Industry Co., Ltd. (Tokyo, Japan). All measurements were extracted in duplicate and read in duplicate (*n* = 4).

### 4.6. Determination of TPC

Reagents, including 2 N Folin–Ciocalteu phenol reagent, Trolox (97%), and catechin, were purchased from Fujifilm Wako Pure Chemical Industries, Ltd. (Osaka, Japan). The TPC was quantified using a modified Folin–Ciocalteu method based on the protocols of Goldstein and Swain [58] and Katsube et al. [59]. Briefly, an aliquot (90 µL) of the prepared extract was transferred into a well of a 96-well microplate (Falcon-3072, Becton–Dickinson, Lincoln Park, NJ, USA). Subsequently, 90 µL of Folin–Ciocalteu reagent and 90 µL of 10% sodium carbonate solution were added to the sample. The mixture was incubated at 25 °C for 60 min to allow color development. The absorbance was then measured at 690 nm using a microplate reader (SH-9000Lab, Corona Electric Co., Ltd., Ibaraki, Japan). Results were calculated using a calibration curve of MR and expressed as mg MR equivalents per 100 g of dry weight (mg MR eq./100 g DW). All measurements were conducted in duplicate extractions with triplicate readings (*n* = 6).

### 4.7. H-ORAC Assay

The H-ORAC assay was performed according to the protocol described by Watanabe et al. with minor modifications [60]. Reagents, including 2,2′-azobis(2-amidinopropane) dihydrochloride (AAPH, 95%) and fluorescein sodium salt, were purchased from Wako Pure Chemical Industries, Ltd. (Osaka, Japan) and Sigma-Aldrich (Steinheim, Germany), respectively. Prior to analysis, sample extracts were diluted with 75 mM phosphate buffer (pH 7.4) and filtered through a 0.45 µm membrane filter (Advantec Toyo Kaisha Ltd., Tokyo, Japan). An aliquot (35 µL) of the diluted sample or Trolox standard was mixed with 115 µL of fluorescein solution (110.7 nmol/L) in a 96-well microplate. The mixture was incubated at 37 °C for 10 min. Subsequently, the oxidation reaction was initiated by adding 50 µL of AAPH solution (31.7 mM). Fluorescence decay was monitored using a fluorescence microplate reader (SH-9000Lab) at 37 °C, with excitation and emission wavelengths set at 485 nm and 520 nm, respectively. Readings were recorded every 2 min over a period of 90 min. The H-ORAC values were calculated based on the net area under the curve (AUC) and expressed as micromoles of Trolox equivalents (TE) per gram of dry weight (µmol TE/g DW). Measurements were performed in duplicate extractions with duplicate readings (*n* = 4).

### 4.8. ABTS Radical Scavenging Assay

The ABTS radical scavenging activity was evaluated based on the method reported by Aimone et al. [61] with slight modifications. Reagents, including 2,2′-azino-bis (3-ethylbenzothiazoline-6-sulfonic acid) (ABTS) and potassium peroxodisulfate, were purchased from Fujifilm Wako Pure Chemical Industries, Ltd. (Osaka, Japan). The ABTS radical cation (ABTS) was generated by mixing 5 mL of 7 mM ABTS aqueous solution with 88 µL of 140 mM potassium peroxodisulfate. The mixture was allowed to react in the dark at room temperature for 12–16 h. Prior to the assay, the radical solution was diluted with 99.5% EtOH to adjust its absorbance to 0.70 ± 0.02 at 734 nm. For the measurement, 35 µL of the sample extract was added to 265 µL of the diluted ABTS working solution. The mixture was agitated for 10 s and subsequently incubated at 30 °C for 4 min. The absorbance was then recorded at 734 nm. Results were expressed as Trolox equivalents (µmol TE/g DW). All analyses were performed in duplicate extractions with triplicate readings (*n* = 6).

### 4.9. Determination of Total Ascorbic Acid Content

Reagents, including L (+)-ascorbic acid, dithiothreitol (DTT), and metaphosphoric acid, were purchased from Fujifilm Wako Pure Chemical Industries, Ltd. (Osaka, Japan). The T-AsA content was determined based on the method described by Lykkesfeldt [62] and Odriozola-Serrano et al. [63] with minor modifications. For the extraction, 40 mL of 2% metaphosphoric acid was added to 200 mg of the sample powder. The mixture was allowed to stand for 1 h to facilitate extraction. Subsequently, the extract was adjusted to a final volume of 50 mL. To quantify the total ascorbic acid (T-AsA), DTT was added to the solution to reduce dehydroascorbic acid to ascorbic acid. The treated sample was then filtered through a 0.45 µm membrane filter (Advantec Toyo Kaisha Ltd., Tokyo, Japan) and subjected to HPLC analysis. Chromatographic separation was performed using a Hitachi LaChrom HPLC system equipped with an InertSustain Swift C18 column (4.6 × 150 mm, 5 µm; GL Sciences Inc., Tokyo, Japan) maintained at 40 °C. The mobile phase consisted of 2% metaphosphoric acid, delivered at a flow rate of 1.0 mL/min. The detection wavelength was set at 245 nm. All measurements were performed in duplicate extractions with duplicate readings (*n* = 4).

### 4.10. UHPLC-ESI-MS/MS Analysis of Flavonoids

To elucidate the chemical structures of the major peaks observed in HPLC, qualitative analysis was performed using ultra-high-performance liquid chromatography coupled with electrospray ionization tandem mass spectrometry (UHPLC-ESI-MS/MS). The analytical conditions were set based on specific modifications to a previous protocol [19,30].

The instrument setup consisted of a Nexera UHPLC system (Shimadzu, Kyoto, Japan) interfaced with a microTOF-QII quadrupole-time-of-flight mass spectrometer (Bruker Daltonik, Bremen, Germany). Chromatographic separation was achieved on an InertSustain Swift C18 column (2.1 mm × 150 mm, GL Sciences, Tokyo, Japan). The column oven temperature was maintained at 40 °C. The mobile phases comprised (A) 0.1% formic acid in water and (B) 0.1% formic acid in acetonitrile. The flow rate was set to 0.3 mL/min, and the injection volume was 2 µL. The gradient elution was programmed as follows: 0–5 min, isocratic hold at 3% B; 5–20 min, linear increase to 50% B; 20–25 min, hold at 50% B; 25–25.1 min, return to initial conditions (3% B); and 25.1–33 min, re-equilibration at 3% B.

Mass spectrometry was conducted in positive electrospray ionization (ESI) mode. The source parameters were configured as follows: capillary voltage, 4500 V; nebulizer pressure, 1.6 bar; dry gas flow, 8.0 L/min; and dry heater temperature, 180 °C. Ion transfer and tuning settings included: Funnel 1 RF, 150 Vpp; Funnel 2 RF, 200 Vpp; hexapole RF, 200 Vpp; quadrupole ion energy, 5 eV; collision energy, 10 eV; collision RF, 500 Vpp; transfer time, 98.4 µs; and pre-pulse storage, 1 µs. Data acquisition was controlled by otofControl v3.2 and HyStar v3.2 software (Bruker Daltonik).

The analysis utilized a data-dependent acquisition (DDA) mode. The top three most intense precursor ions were selected for collision-induced dissociation (CID) fragmentation with a switching threshold of 5000 counts. Active exclusion was applied after two spectra and released after 1 min. The mass scanning range was set to *m*/*z* 50–1000 for both survey and MS/MS spectra (acquisition rate: 1 Hz), excluding precursors in the ranges of *m*/*z* 50–200 and 800–1500.

### 4.11. Analysis of Mineral Composition

The mineral composition of the samples was determined using inductively coupled plasma mass spectrometry (ICP-MS) coupled with a wet digestion method, following previously established procedures [64,65] with minor modifications. Reagents, including nitric acid (HNO_3_) and hydrogen peroxide (H_2_O_2_), were purchased from Kanto Chemical Co., Inc. (Tokyo, Japan). For sample preparation, 0.5 g of the powdered leaf material was placed in a digestion vessel and mixed with 10 mL of HNO_3_, 2 mL of H_2_O_2_, and 5 mL of distilled water. The mixture was digested at 200 °C for 20 min using an ECOPRE-II digestion system (ODLAB, Gwangmyeong-si, Republic of Korea; distributed by Actac, Tokyo, Japan). After cooling, the digested solution was diluted with distilled water to an appropriate volume. The concentrations of mineral elements were then quantified using an Agilent 8800 ICP-MS system (Agilent Technologies, Hanover, Germany). All measurements were performed in triplicate for each sample (*n* = 3).

### 4.12. Analysis of Volatile Compounds via GC-MS

The profile of volatile components was characterized using headspace solid-phase microextraction (HS-SPME) coupled with gas chromatography–mass spectrometry (GC-MS), based on previously reported methods [13] with minor modifications. For the analysis, 0.5 g of the pulverized powder from raw materials (RB, NL, and OL) was placed directly into a 20 mL headspace vial. For the processed leaf teas (FD, S-MD, MD, and SD), 0.5 g of the sample powder was mixed with 5 mL of deionized water in the vial to simulate infusion conditions. The vials were sealed and pre-incubated at 60 °C for 5 min to ensure equilibrium. Extraction was performed by exposing a 50/30 µm divinylbenzene/Carboxen/polydimethylsiloxane (DVB/CAR/PDMS) fiber (Sigma-Aldrich, Tokyo, Japan) to the headspace at 60 °C for 30 min. Following extraction, the fiber was inserted into the injection port of a GCMS-QP2020 single quadrupole mass spectrometer equipped with an AOC-6000 autosampler (Shimadzu, Kyoto, Japan). Desorption was carried out at 250 °C in split mode for 1 min. Chromatographic separation was achieved on a DB-HeavyWAX column (60 m × 0.25 mm, 0.25 µm film thickness; Agilent Technologies, Santa Clara, CA, USA) using helium as the carrier gas at a constant pressure of 150 kPa. The oven temperature program was set as follows: initial hold at 50 °C for 4 min, followed by a ramp of 5 °C/min to 250 °C, where it was maintained for 15 min. The mass spectrometer was operated with an electron ionization (EI) source at 70 eV. The ion source and transfer line temperatures were set to 200 °C and 250 °C, respectively. Mass spectra were acquired in the scanning range of *m*/*z* 30–400. Volatile compounds were tentatively identified by comparing their mass spectral patterns with those in the NIST/EPA/NIH Mass Spectral Library (NIST 17). All measurements were performed in triplicate for each sample (*n* = 3).

### 4.13. Statistical Analysis

All statistical computations were performed using the IBM SPSS Statistics software package (Version 28; IBM Corp., Chicago, IL, USA). To evaluate differences between group means, data were subjected to one-way analysis of variance (ANOVA) followed by Tukey’s honest significant difference (HSD) test for multiple comparisons. A *p*-value of less than 0.05 (*p* < 0.05) was considered statistically significant. All quantitative results are presented as the mean ± standard error (SE).

## 5. Conclusions

This study comprehensively evaluated the changes in phytochemical profiles, antioxidant capacities, and volatile compositions of BL across different growth stages and processing methods. Regarding the harvest timing, although RB exhibited the highest abundance of volatiles and antioxidants, NL was identified as the optimal raw material for industrial applications due to the favorable balance between bioactive content and biomass availability. In terms of processing, a distinct trade-off was elucidated between nutrient retention and extraction efficiency. FD proved superior in preserving heat-sensitive ascorbic acid, fresh volatile compounds (monoterpenes), and the vibrant green color of the leaves. Conversely, thermal drying methods, particularly S-MD, significantly enhanced the extraction efficiency of polyphenols (such as MR) and minerals by disrupting cellular barriers. Evaluation of the tea infusions prepared from the processed leaf powders by hot water extraction to simulate traditional tea brewing revealed that S-MD- and MD-processed powders yielded the highest antioxidant activity, as measured by the H-ORAC assay, demonstrating their superior suitability as functional tea ingredients. SD resulted in the lowest overall quality, as the prolonged drying duration permitted endogenous enzymatic activity, leading to substantial degradation of both phenolics and chlorophyll. Consequently, the selection of the drying method should be dictated by the target product application: FD is ideal for high-value teas where aesthetics and fresh aroma are prioritized, whereas S-MD is recommended for developing cost-effective, antioxidant-rich functional food ingredients. These findings provide a scientific basis for the valorization of BL, both as a functional tea ingredient and as a broader health-promoting resource, contributing to the sustainable utilization of this largely unexploited biomass.

## Data Availability

The original contributions presented in the study are included in the article; further inquiries can be directed to the corresponding author.

## Abbreviations

The following abbreviations are used in this manuscript:

AAPH	2,2′-Azobis(2-amidinopropane) dihydrochloride
ABTS	2,2′-Azino-bis(3-ethylbenzothiazoline-6-sulfonic acid)
BL	Bayberry leaves
DPPH	2,2-Diphenyl-1-picrylhydrazyl
DTT	Dithiothreitol
DW	Dry weight
(E)GC	(epi)gallocatechin
(E)GCG	(epi)gallocatechin gallate
EtOH	Ethanol
FD	Freeze-drying
GC-MS	Gas chromatography-mass spectrometry
H-ORAC	Hydrophilic oxygen radical absorbance capacity
HPLC	High-performance liquid chromatography
HS-SPME	Headspace solid-phase microextraction
HW	Hot water
ICP-MS	Inductively coupled plasma mass spectrometry
MD	Mechanical drying
MR	Myricitrin
NL	New leaves
OL	Old leaves
RB	Red buds
RT	Retention time
SD	Shade drying
SEM	Scanning electron microscopy
S-MD	Steaming followed by mechanical drying
T-AsA	Total ascorbic acid
TE	Trolox equivalents
TIC	Total ion chromatograms
TPC	Total phenolic content
UHPLC-ESI-MS/MS	Ultra-high-performance liquid chromatography-electrospray ionization tandem mass spectrometry
W	Water

## References

[1] Ren H., Yu H., Zhang S., Liang S., Zheng X., Zhang S., Yao P., Zheng H., Qi X. (2019). Genome sequencing provides insights into the evolution and antioxidant activity of Chinese bayberry. BMC Genom..

[2] Silva B.J., Seca A.M., Barreto M.d.C., Pinto D.C. (2015). Recent breakthroughs in the antioxidant and anti-inflammatory effects of *Morella* and *Myrica* species. Int. J. Mol. Sci..

[3] Zhang S., Yu Z., Sun L., Ren H., Zheng X., Liang S., Qi X. (2022). An overview of the nutritional value, health properties, and future challenges of Chinese bayberry. PeerJ.

[4] Yan S., Zhang X., Wen X., Lv Q., Xu C., Sun C., Li X. (2016). Purification of flavonoids from Chinese bayberry (*Morella rubra* Sieb. et Zucc.) fruit extracts and α-glucosidase inhibitory activities of different fractionations. Molecules.

[5] Liu Z., Zhang J., Lu S., Tang W., Zhou Y., Quek S.Y. (2022). Effects of different drying methods on phenolic components and in vitro hypoglycemic activities of pulp extracts from two Chinese bayberry (*Myrica rubra* Sieb. et Zucc.) cultivars. Food Sci. Hum. Wellness.

[6] Ju J., Yao W., Sun S., Guo Y., Cheng Y., Qian H., Xie Y. (2018). Assessment of the antibacterial activity and the main bacteriostatic components from bayberry fruit extract. Int. J. Food Prop..

[7] Zhang Y., Chen S., Wei C., Rankin G.O., Ye X., Chen Y.C. (2018). Dietary compound proanthocyanidins from Chinese bayberry (*Myrica rubra* Sieb. et Zucc.) leaves attenuate chemotherapy-resistant ovarian cancer stem cell traits via targeting the Wnt/β-catenin signaling pathway and inducing G1 cell cycle arrest. Food Funct..

[8] Zhang Y., Chen S., Wei C., Gong H., Li L., Ye X. (2016). Chemical and Cellular Assays Combined with In Vitro Digestion to Determine the Antioxidant Activity of Flavonoids from Chinese Bayberry (*Myrica rubra Sieb.* et Zucc.) Leaves. PLoS ONE.

[9] Meyer E., Mori M.A., Campos A.C., Andreatini R., Guimarães F.S., Milani H., de Oliveira R.M.W. (2017). Myricitrin induces antidepressant-like effects and facilitates adult neurogenesis in mice. Behav. Brain Res..

[10] Pereira M., Siba I.P., Acco A., Correia D., Lapa F.R., Santos A.R., Ruani A.P., Pizzolatti M.G., Andreatini R. (2022). Myricitrin exhibits antidepressant-like effects and reduces IL-6 hippocampal levels in the chronic mild stress model. Behav. Brain Res..

[11] Fernandez S.P., Nguyen M., Yow T.T., Chu C., Johnston G.A., Hanrahan J.R., Chebib M. (2009). The flavonoid glycosides, myricitrin, gossypin and naringin exert anxiolytic action in mice. Neurochem. Res..

[12] Haw Y.T., Sim Y.Y., Nyam K.L. (2020). Effect of steam blanching and high temperature drying on the physicochemical properties, antioxidant activities and consumer acceptability of *Hibiscus cannabinus* leaves tea. J. Food Sci. Technol..

[13] Qu F.F., Li X.H., Wang P.Q., Han Y.H., Wu Y., Hu J.H., Zhang X.F. (2023). Effect of thermal process on the key aroma components of green tea with chestnut-like aroma. J. Sci. Food Agric..

[14] Sun C., Huang H., Xu C., Li X., Chen K. (2013). Biological activities of extracts from Chinese bayberry (*Myrica rubra* Sieb. et Zucc.): A review. Plant Foods Hum. Nutr..

[15] Kubick D.C., Owen T.P. (2003). Bulletin No. 38: The Hidden World of Plants: A Scanning Electron Microscope Survey of the Native Plant Collection, Connecticut College Arboretum. https://digitalcommons.conncoll.edu/cgi/viewcontent.cgi?article=1038&context=arbbulletins.

[16] Turkmen N., Sari F., Velioglu Y.S. (2006). Effects of extraction solvents on concentration and antioxidant activity of black and black mate tea polyphenols determined by ferrous tartrate and Folin–Ciocalteu methods. Food Chem..

[17] Frempong T.F., Boadi N.O., Badu M. (2021). Optimization of extraction conditions for polyphenols from the stem bark of *Funtumia elastica* (Funtum) utilizing response surface methodology. AAS Open Res..

[18] Lee S.-G., Lee D., Kang H. (2020). Effect of Extraction Ethanol Concentration on Antioxidant and Anti-Inflammatory Activity of 30-Year-Old and 120-Year-Old Dangyuja (*Citrus maxima* (Burm.) Merr.). Biomed. Sci. Lett..

[19] Yang H., Ge Y., Sun Y., Liu D., Ye X., Wu D. (2011). Identification and characterisation of low-molecular-weight phenolic compounds in bayberry (*Myrica rubra* Sieb. et Zucc.) leaves by HPLC-DAD and HPLC-UV-ESIMS. Food Chem..

[20] Dai J., Mumper R.J. (2010). Plant phenolics: Extraction, analysis and their antioxidant and anticancer properties. Molecules.

[21] Spigno G., Tramelli L., De Faveri D.M. (2007). Effects of extraction time, temperature and solvent on concentration and antioxidant activity of grape marc phenolics. J. Food Eng..

[22] Cacace J., Mazza G. (2003). Mass transfer process during extraction of phenolic compounds from milled berries. J. Food Eng..

[23] Zou M., Tao W., Ye X., Liu D. (2019). Evaluation of antimicrobial and antibiofilm properties of proanthocyanidins from Chinese bayberry (*Myrica rubra* Sieb. et Zucc.) leaves against Staphylococcus epidermidis. Food Sci. Nutr..

[24] Yang H., Ye X., Liu D., Chen J., Zhang J., Shen Y., Yu D. (2011). Characterization of unusual proanthocyanidins in leaves of bayberry (*Myrica rubra Sieb*. et Zucc.). J. Agric. Food Chem..

[25] Prior R.L., Wu X., Schaich K. (2005). Standardized methods for the determination of antioxidant capacity and phenolics in foods and dietary supplements. J. Agric. Food Chem..

[26] Ministry of Health, Labour and Welfare (2020). The Dietary Reference Intakes for Japanese (2020).

[27] Shen S., Zhao M., Li C., Chang Q., Liu X., Liao Y., Pan R. (2019). Study on the material basis of neuroprotection of *Myrica rubra* bark. Molecules.

[28] Li J., Wang H., Li J., Liu Y., Ding H. (2020). LC-MS analysis of *Myrica rubra* extract and its hypotensive effects via the inhibition of GLUT 1 and activation of the NO/Akt/eNOS signaling pathway. RSC Adv..

[29] Liu Y., Zhang X., Zhan L., Xu C., Sun L., Jiang H., Sun C., Li X. (2020). LC-Q-TOF-MS characterization of polyphenols from white bayberry fruit and its antidiabetic effect in KK-Ay mice. ACS Omega.

[30] Cuyckens F., Claeys M. (2004). Mass spectrometry in the structural analysis of flavonoids. J. Mass Spectrom..

[31] Yu J., Zhao X., He Y., Zhang Y., Tang C. (2025). An Innovative Strategy for Untargeted Mass Spectrometry Data Analysis: Rapid Chemical Profiling of the Medicinal Plant Terminalia chebula Using Ultra-High-Performance Liquid Chromatography Coupled with Q/TOF Mass Spectrometry–Key Ion Diagnostics–Neutral Loss Filtering. Molecules.

[32] Spínola V., Llorent-Martínez E.J., Gouveia S., Castilho P.C. (2014). *Myrica faya*: A new source of antioxidant phytochemicals. J. Agric. Food Chem..

[33] Fu Y., Qiao L., Cao Y., Zhou X., Liu Y., Ye X. (2014). Structural elucidation and antioxidant activities of proanthocyanidins from Chinese bayberry (*Myrica rubra* Sieb. et Zucc.) leaves. PLoS ONE.

[34] Taamalli A., Iswaldi I., Arráez-Román D., Segura-Carretero A., Fernández-Gutiérrez A., Zarrouk M. (2014). UPLC–QTOF/MS for a Rapid Characterisation of Phenolic Compounds from Leaves of *Myrtus communis* L.. Phytochem. Anal..

[35] Shakeel F., Haq N., Alshehri S., Ibrahim M.A., Elzayat E.M., Altamimi M.A., Mohsin K., Alanazi F.K., Alsarra I.A. (2018). Solubility, thermodynamic properties and solute-solvent molecular interactions of luteolin in various pure solvents. J. Mol. Liq..

[36] Parveen S., Bhat I.U.H., Bhat R. (2023). Kaempferol and its derivatives: Biological activities and therapeutic potential. Asian Pac. J. Trop. Biomed..

[37] Özbek H., Sever Yilmaz B. (2017). Anti-inflammatory and hypoglycemic activities of alpha-pinene. Acta Pharm. Sci..

[38] Mollica F., Gelabert I., Amorati R. (2022). Synergic antioxidant effects of the essential oil component γ-terpinene on high-temperature oil oxidation. ACS Food Sci. Technol..

[39] Ramalho T.R.O. (2015). Gamma-Terpinene Modulates Acute Inflammatory Response in Mice. Planta Med..

[40] Lanciotti R., Belletti N., Patrignani F., Gianotti A., Gardini F., Guerzoni M.E. (2003). Application of hexanal,(*E*)-2-hexenal, and hexyl acetate to improve the safety of fresh-sliced apples. J. Agric. Food Chem..

[41] Balahbib A., El Omari N., Hachlafi N.E., Lakhdar F., El Menyiy N., Salhi N., Mrabti H.N., Bakrim S., Zengin G. (2021). Health beneficial and pharmacological properties of p-cymene. Food Chem. Toxicol..

[42] Li Q., Zhu X., Xie Y., Liang J. (2021). Antifungal properties and mechanisms of three volatile aldehydes (octanal, nonanal and decanal) on *Aspergillus flavus*. Grain Oil Sci. Technol..

[43] Okhale S.E., Ugbabe G.E., Oladosu P.O., Ibrahim J.A., Egharevba H.O., Kunle O.F., Elisha E.P., Chibuike A.J., Ezem S.U. (2018). Chemical constituents and antimicrobial activity of the leaf essential oil of *Ixora coccinea* L. (Rubiaceae) collected from North Central Nigeria. Int. J. Bioassays.

[44] Guo Y., Zhang T., Zhong J., Ba T., Xu T., Zhang Q., Sun M. (2020). Identification of the volatile compounds and observation of the glandular trichomes in *Opisthopappus taihangensis* and four species of *Chrysanthemum*. Plants.

[45] Chen J.Y., Kuruparan A., Zamani-Babgohari M., Gonzales-Vigil E. (2023). Dynamic changes to the plant cuticle include the production of volatile cuticular wax–derived compounds. Proc. Natl. Acad. Sci. USA.

[46] Guo J., Yuan Y., Liu Z., Zhu J. (2013). Development and structure of internal glands and external glandular trichomes in *Pogostemon cablin*. PLoS ONE.

[47] Zhou X., Chen X., Du Z., Zhang Y., Zhang W., Kong X., Thelen J.J., Chen C., Chen M. (2019). Terpenoid esters are the major constituents from leaf lipid droplets of Camellia sinensis. Front. Plant Sci..

[48] Wang L., Jäggi S., Cofer T.M., Waterman J.M., Walthert M., Glauser G., Erb M. (2023). Immature leaves are the dominant volatile-sensing organs of maize. Curr. Biol..

[49] Nowak D., Jakubczyk E. (2020). The freeze-drying of foods—The characteristic of the process course and the effect of its parameters on the physical properties of food materials. Foods.

[50] Koca İ., Lüle F., Koyuncu T. (2018). Effect of microwave and hot-air drying techniques on the color properties and specific energy requirement of madimak plants (*Polygonum cognatum* Meissn.). J. Biol. Environ. Sci..

[51] Li R., Shang H., Wu H., Wang M., Duan M., Yang J. (2018). Thermal inactivation kinetics and effects of drying methods on the phenolic profile and antioxidant activities of chicory (*Cichorium intybus* L.) leaves. Sci. Rep..

[52] Chen Y., Jiang Z., Wu S., Cheng B., Zhou L., Liu T., Yu C. (2025). Structure and release function of fragrance glands. Hortic. Res..

[53] Chen M. (2021). The tea plant leaf cuticle: From plant protection to tea quality. Front. Plant Sci..

[54] Krakowska-Sieprawska A., Kiełbasa A., Rafińska K., Ligor M., Buszewski B. (2022). Modern methods of pre-treatment of plant material for the extraction of bioactive compounds. Molecules.

[55] Hazrati S., Lotfi K., Govahi M., Ebadi M.T. (2021). A comparative study: Influence of various drying methods on essential oil components and biological properties of *Stachys lavandulifolia*. Food Sci. Nutr..

[56] Wang X., Zeng L., Liao Y., Zhou Y., Xu X., Dong F., Yang Z. (2019). An alternative pathway for the formation of aromatic aroma compounds derived from L-phenylalanine via phenylpyruvic acid in tea (*Camellia sinensis* (L.) O. Kuntze) leaves. Food Chem..

[57] Supriyadi S., Nareswari A.R., Fitriani A., Gunadi R. (2021). Enhancement of Black Tea Aroma by Adding the β-Glucosidase Enzyme during Fermentation on Black Tea Processing. Int. J. Food Sci..

[58] Goldstein J.L., Swain T. (1963). Changes in tannins in ripening fruits. Phytochemistry.

[59] Katsube T., Tabata H., Ohta Y., Yamasaki Y., Anuurad E., Shiwaku K., Yamane Y. (2004). Screening for antioxidant activity in edible plant products: Comparison of low-density lipoprotein oxidation assay, DPPH radical scavenging assay, and Folin–Ciocalteu assay. J. Agric. Food Chem..

[60] Watanabe J., Oki T., Takebayashi J., Yamasaki K., Takano-Ishikawa Y., Hino A., Yasui A. (2012). Method validation by interlaboratory studies of improved hydrophilic oxygen radical absorbance capacity methods for the determination of antioxidant capacities of antioxidant solutions and food extracts. Anal. Sci..

[61] Aimone C., Grillo G., Boffa L., Giovando S., Cravotto G. (2023). Tannin extraction from chestnut wood waste: From lab scale to semi-industrial plant. Appl. Sci..

[62] Lykkesfeldt J. (2000). Determination of ascorbic acid and dehydroascorbic acid in biological samples by high-performance liquid chromatography using subtraction methods: Reliable reduction with tris [2-carboxyethyl] phosphine hydrochloride. Anal. Biochem..

[63] Odriozola-Serrano I., Hernández-Jover T., Martín-Belloso O. (2007). Comparative evaluation of UV-HPLC methods and reducing agents to determine vitamin C in fruits. Food Chem..

[64] Nardi E.P., Evangelista F.S., Tormen L., Saint T.D., Curtius A.J., de Souza S.S., Barbosa F. (2009). The use of inductively coupled plasma mass spectrometry (ICP-MS) for the determination of toxic and essential elements in different types of food samples. Food Chem..

[65] Li Y., Zhang D., Huang Y., Yuan S., Xu Z. (2015). Speciation and Optimization of Multi-Elements Analysis of River Sediment in Shanghai by ICP-MS with Microwave-Assisted Digestion Method. Asian J. Chem..

